# Intraoperative electrical stimulation of the human dorsal spinal cord reveals a map of arm and hand muscle responses

**DOI:** 10.1152/jn.00235.2022

**Published:** 2022-11-23

**Authors:** James R. McIntosh, Evan F. Joiner, Jacob L. Goldberg, Lynda M. Murray, Bushra Yasin, Anil Mendiratta, Steven C. Karceski, Earl Thuet, Oleg Modik, Evgeny Shelkov, Joseph M. Lombardi, Zeeshan M. Sardar, Ronald A. Lehman, Christopher Mandigo, K. Daniel Riew, Noam Y. Harel, Michael S. Virk, Jason B. Carmel

**Affiliations:** ^1^Department of Orthopedic Surgery, Columbia University, New York, New York; ^2^Department of Neurological Surgery, Columbia University, New York, New York; ^3^Department of Neurology, Columbia University, New York, New York; ^4^Department of Neurological Surgery, Weill Cornell Medicine-New York Presbyterian, Och Spine Hospital, New York, New York; ^5^Department of Neurology, Weill Cornell Medicine-New York Presbyterian, Och Spine Hospital, New York, New York; ^6^New York Presbyterian, Och Spine Hospital, New York, New York; ^7^Department of Neurology, Icahn School of Medicine at Mount Sinai, New York, New York; ^8^Department of Rehabilitation and Human Performance, Icahn School of Medicine at Mount Sinai, New York, New York; ^9^James J. Peters Veterans Affairs Medical Center, Bronx, New York

**Keywords:** cervical spinal cord injury, electrical stimulation, epidural, motor evoked potentials, myelopathy

## Abstract

Although epidural stimulation of the lumbar spinal cord has emerged as a powerful modality for recovery of movement, how it should be targeted to the cervical spinal cord to activate arm and hand muscles is not well understood, particularly in humans. We sought to map muscle responses to posterior epidural cervical spinal cord stimulation in humans. We hypothesized that lateral stimulation over the dorsal root entry zone would be most effective and responses would be strongest in the muscles innervated by the stimulated segment. Twenty-six people undergoing clinically indicated cervical spine surgery consented to mapping of motor responses. During surgery, stimulation was performed in midline and lateral positions at multiple exposed segments; six arm and three leg muscles were recorded on each side of the body. Across all segments and muscles tested, lateral stimulation produced stronger muscle responses than midline despite similar latency and shape of responses. Muscles innervated at a cervical segment had the largest responses from stimulation at that segment, but responses were also observed in muscles innervated at other cervical segments and in leg muscles. The cervical responses were clustered in rostral (C4–C6) and caudal (C7–T1) cervical segments. Strong responses to lateral stimulation are likely due to the proximity of stimulation to afferent axons. Small changes in response sizes to stimulation of adjacent cervical segments argue for local circuit integration, and distant muscle responses suggest activation of long propriospinal connections. This map can help guide cervical stimulation to improve arm and hand function.

**NEW & NOTEWORTHY** A map of muscle responses to cervical epidural stimulation during clinically indicated surgery revealed strongest activation when stimulating laterally compared to midline and revealed differences to be weaker than expected across different segments. In contrast, waveform shapes and latencies were most similar when stimulating midline and laterally, indicating activation of overlapping circuitry. Thus, a map of the cervical spinal cord reveals organization and may help guide stimulation to activate arm and hand muscles strongly and selectively.

## Graphical abstract

**Figure:**
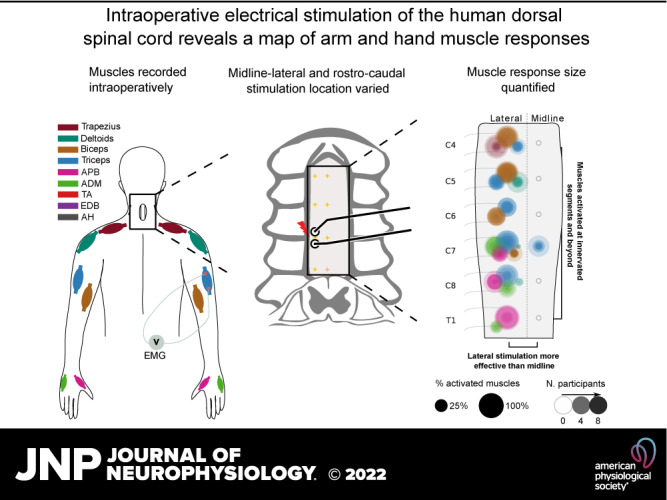

## INTRODUCTION

Epidural stimulation has emerged as a way to modify spinal cord circuits for movement recovery (1–5). These studies have largely targeted the lumbar spinal cord, with its relatively well-defined central pattern generator (6, 7). In contrast, the circuit-level logic of where and how to stimulate the cervical spinal cord is not as well known. Since hand function is the top priority of people with cervical spinal cord injury (SCI) (8, 9), interventions are under development to target the cervical spinal cord (10–15). Effective stimulation of the cervical spinal cord may be more difficult than for the lumbosacral spinal cord given its large behavioral repertoire and poorly understood intrinsic programs (16). The present study used electrical stimulation during clinically indicated cervical spine surgery to improve understanding of cervical spinal cord circuits.

Some of the insights learned from lumbar epidural stimulation apply to the cervical spinal cord. Stimulation of the dorsal spinal cord at low intensity evokes muscle responses via large-diameter afferents and not by direct activation of motoneurons (17, 18). Electrical stimulation of afferents activates motoneurons through circuits involved in reflexes (19, 20). This mechanism has been corroborated by mathematical modeling of current flow (21–23) as well as in inactivation studies (14). Consistent with this model, we have shown that stimulating near the dorsal root entry zone is more effective than stimulating at midline in the cervical spinal cord of rats (24).

Dorsal epidural stimulation of a cervical segment activates muscles innervated at that segment with spread to adjacent segments. In work performed mostly in monkeys, muscle responses to epidural stimulation of the cervical spinal cord (23, 25) broadly correspond to the distribution of motor pools throughout the cervical cord (26, 27). The spread of responses beyond the stimulated segment may be due to spread of afferents (28, 29) or variability across participants in the course of motor axons emanating from a cervical ventral root through the brachial plexus and peripheral nerves (30–32). For example, with the exception of the separation between the biceps and triceps, Greiner et al. (23) observed a considerable overlap in the activated muscles at each cervical spinal segment in macaque monkeys. When stimulating dorsally in humans this breadth of activation of individual muscles across multiple segments is present; however, some additional divergence has also been found (33), with the most extreme observation being of leg muscle activation to dorsal epidural cervical stimulation (34). Leg muscle activation has also been observed in transcutaneous dorsal cervical stimulation (35).

**Figure 1.**
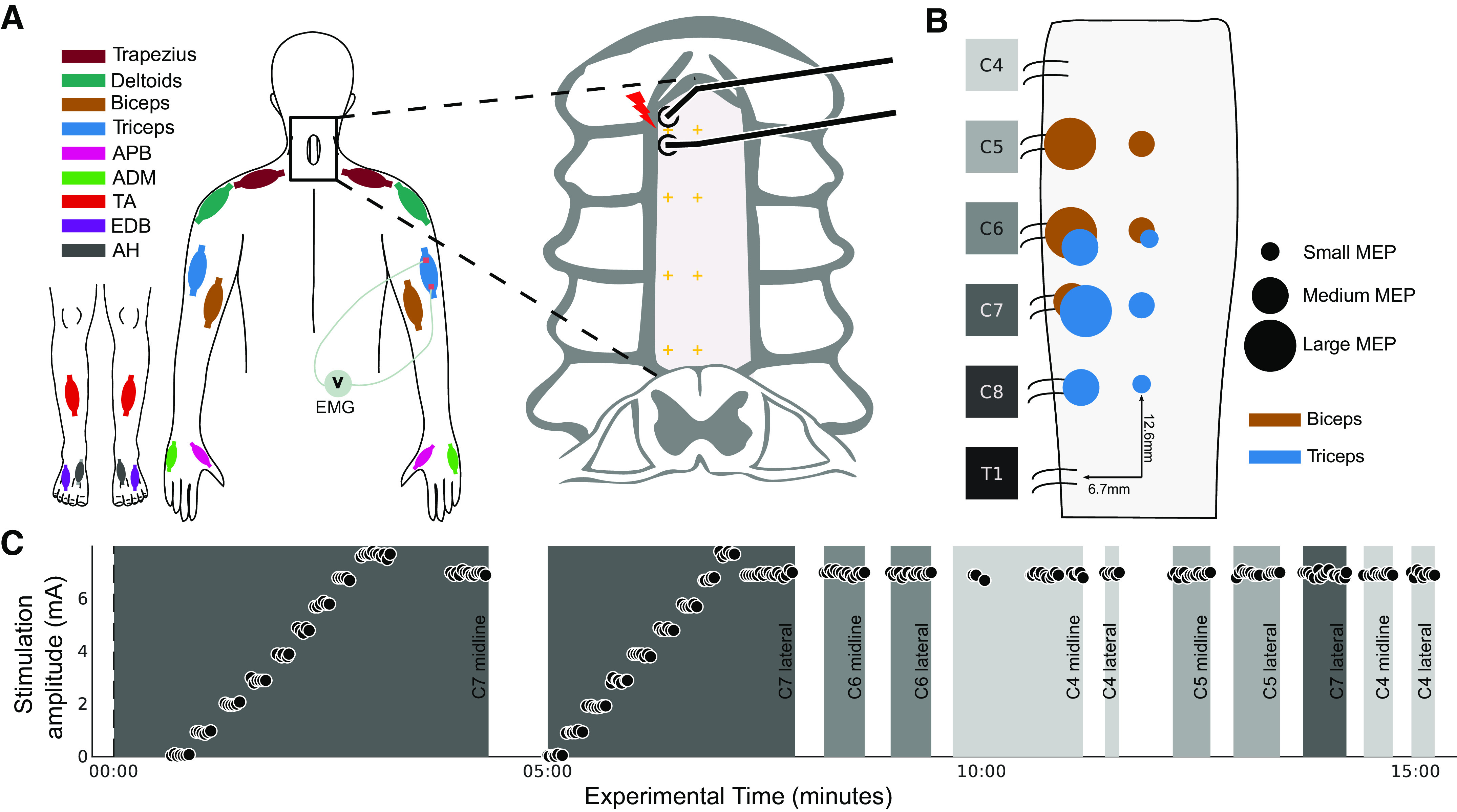
Epidural stimulation experiment during posterior cervical spine surgery. *A*: once the dura was exposed by laminectomy, a handheld or catheter electrode was used to stimulate at multiple segmental locations in either the lateral or midline position. Colors correspond to different recorded muscles (see key). ADM, abductor digiti minimi; AH, abductor hallucis; APB, abductor pollicis brevis; EDB, extensor digitorum brevis; TA, tibialis anterior. *B*: hypothesized responses to epidural stimulation of the cervical cord: *1*) Lateral stimulation over the dorsal root entry zone was expected to be more effective than stimulation over the midline (large circles representing high activation are lateral, and smaller circles are placed along the midline). *2*) Muscle activation was expected to be relatively localized in the rostral-caudal direction; however, some spread of this activation was expected. MEP, motor evoked potential. *C*: example sequence of the 15-min experiment. Initially, the electrode was placed along the midline, over the dura at a segment aligned with the expected location of the root entry (C7 midline for this experiment). The stimulation intensity was ramped up with 2 purposes: *1*) in search of the threshold of a target muscle and *2*) to gather threshold information for multiple muscles in a single sweep. Once the threshold was found, stimulation was performed at 1.2× that value. The procedure was then repeated laterally to enable a midline-lateral comparison. Stimulation was performed at multiple segments at both midline and lateral locations.

These data led us to hypothesize that epidural stimulation of each cervical segment would activate large-diameter afferents, causing strongest contraction of muscles innervated at that segment when stimulating laterally near the dorsal root entry zone. The detailed predicted results are shown in Fig. 1*B*, with larger circles representing larger motor evoked potentials (MEPs) provoked by stimulation at that location in biceps (brown) or triceps (blue). Similar to our studies in rats (24), we predicted that lateral stimulation of the spinal cord would be more effective than midline stimulation. The strongest responses would be observed at the segment of innervation [e.g., C5 and C6 for biceps (36) and C7 for triceps] with spread from there to adjacent segments. Taken together, we expected that there would be a larger change in the size of motor evoked potentials when comparing midline to lateral stimulation within each segment than when comparing rostral-caudal cervical segments. Despite these differences, we expected that midline and lateral stimulation would activate similar circuits, and we tested this by comparing MEP waveform shape and onset latency. The results are expected to help target cervical epidural stimulation to activate or modulate arm and hand muscles.

## MATERIALS AND METHODS

### Experimental Design

In people undergoing clinically indicated spine surgery, epidural electrical stimulation of the exposed segments of the cervical spinal cord was performed and motor evoked potentials (MEPs) from arm and leg muscles were recorded (Fig. 1*A*). Surgical time was not extended by the experimental procedure for longer than 15 min to limit the surgical and anesthetic risk of increased intraoperative time (37). To test whether lateral stimulation would be more effective than midline, stimulation at each of these sites was compared (Fig. 1*B*). To test whether stimulation at one cervical segment would produce the largest responses in muscles innervated at that segment, stimulation at multiple cervical roots was performed. To test whether circuits activated at midline and lateral stimulation are more similar to each other than circuits activated at different rostral-caudal segments, we compared MEP onset latencies and correlations in waveform shape. To test whether moving the stimulating electrodes in the midline-lateral direction would produce larger changes than in the rostro-caudal direction, the size of MEP change over change in distance was computed for each direction (see *Comparison of responses at different cervical segments* below in materials and methods). A representative experiment is shown in Fig. 1*C*. The primary outcome was the size of the MEP, and the secondary outcomes were the threshold and slope of the recruitment curve with increasing stimulus intensity, as well as the onset latency and shape of the resultant MEPs.

### Confirmation of Electrode Position over the Dorsal Root Entry Zone

**Figure 2.**
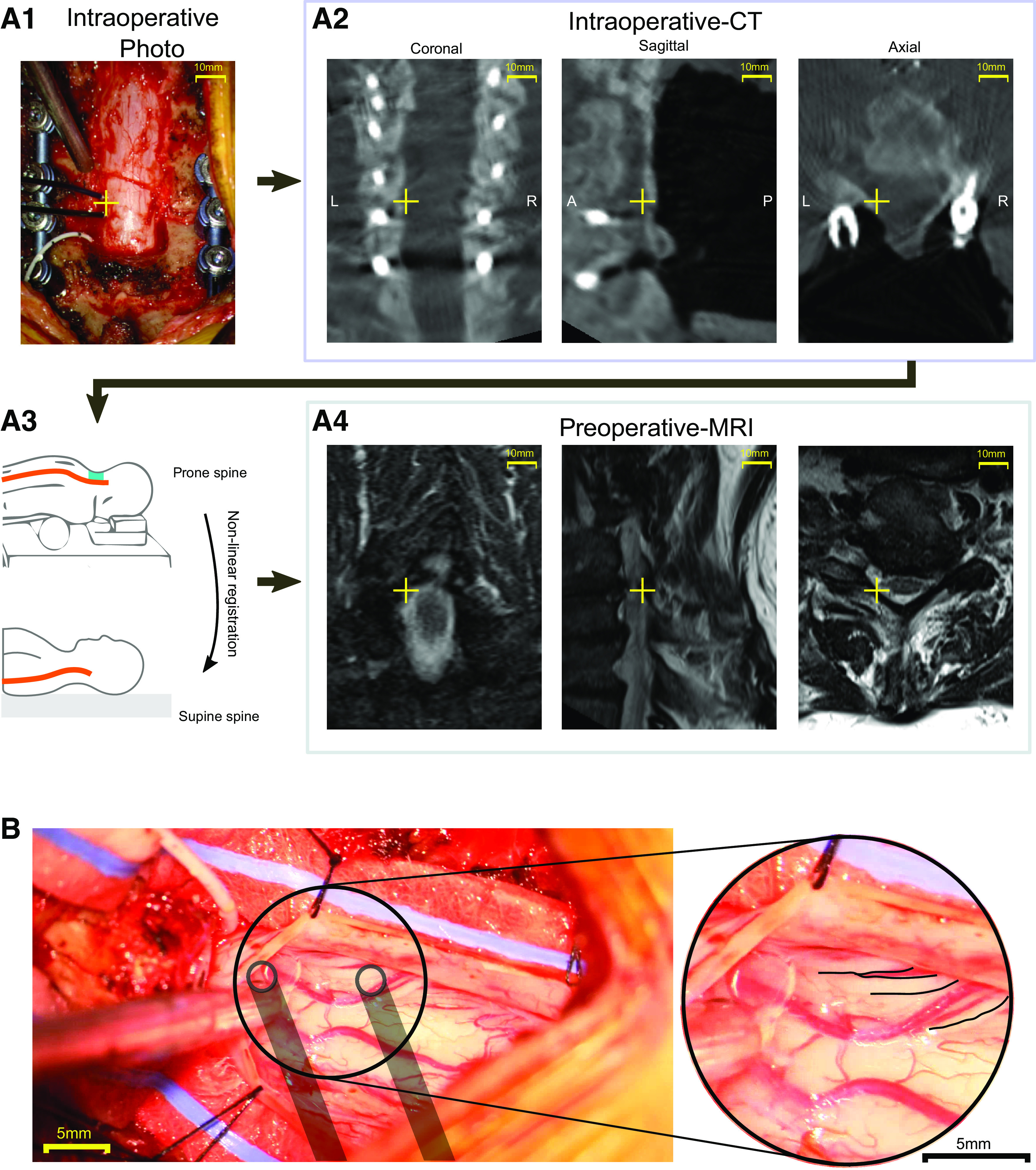
Validation of stimulation location relative to dorsal root entry zones. *A*: transformation of electrode stimulation site from physical location to MRI space. *A1*: a photograph was used to identify the position of electrodes relative to instrumentation. *A2*: this location was translated to a computer tomography (CT) scan used to confirm implant location (yellow crosshairs). *A3*: CT and MRI images were coregistered by performing a nonlinear segmental registration (Brainlab AG) to account for differences in spinal alignment found in prone (operative) and supine (preoperative) positions. *A4*: electrode position in MRI near targeted C7 root verified location. *B*: exposed subdural region with overlaid location of stimulated location and dorsal roots highlighted in *inset*. Placement based on anatomical landmark identification was confirmed to position the electrodes over the dorsal root entry zones.

When placed laterally, electrodes were positioned straddling the dorsal root entry zone in a rostral-caudal orientation. The variability in the distance between the intervertebral foramen and caudal rootlets in the cervical cord has been shown to be small relative to the size of our electrodes (38). Consequently, we identified the expected location of the root exit from the bony and neural anatomical landmarks. Specifically, the exiting cervical nerve was identified at each relevant level as it entered the neural foramen just caudal to the pedicle. The electrodes were then placed at the site where the dorsal root enters the lateral spinal cord. Electrode placement based on anatomical landmarks was subsequently confirmed to be over the dorsal root entry zone via image reconstruction and coregistration between preoperative MRI and intraoperative computer tomography (CT) scans performed in one participant. Clinically indicated intraoperative CT (Airo; Stryker Inc.) and preoperative MRI (T2 weighted; Siemens) were gathered for this participant. During the course of the experiment, the location of the C7 root entry was inferred based on the bony landmarks of the spine. After the electrode was placed at that site an intraoperative photograph was taken (Fig. 2*A1*). The location of the electrode was then translated to a location in the CT based on instrumentation inserted during surgery (Fig. 2*A2*). The CT and MRI images were then coregistered by performing a nonlinear registration with Curvature Correction software (Brainlab AG) to account for the different curvature of the spine in prone (operative) and supine (preoperative) participant positions (Fig. 2*A3*). The transformation generated in the registration was then also applied to the location of the electrode in the CT and shown in the MRI (Fig. 2*A4*).

Confirmation of electrode position was also made visually in an additional experimental condition in which stimulation was applied at the T1 root entry, below the surface of the dura and at matched epidural locations (Fig. 2*B*).

### Participants

Participants were adult patients with cervical spondylotic myelopathy, multilevel foraminal stenosis, or intradural tumor requiring surgical treatment (Table 1, Supplemental Table S1; all Supplemental Material is available at https://doi.org/10.6084/m9.figshare.19891966). Patients were enrolled from the clinical practices of the spine surgeons participating in the study. The study protocol was reviewed and approved by the Institutional Review Boards of the two study sites, Weill Cornell Medical Center and Columbia University Irving Medical Center (ClinicalTrials.gov number: NCT05163639; participants were recruited after registration). Patients with stimulation devices in the neck or chest (e.g., vagal nerve stimulation, cardiac patients with pacemakers) or head and neck implants were excluded. Written informed consent for participation in the study was obtained before surgery for every participant. Participants underwent standard of care preoperative clinical assessments. Clinical MRI scans were used to determine T2 signal hyperintensity within the spinal cord and also the degree of foraminal stenosis. We defined severe foraminal stenosis as no detectable cerebrospinal fluid within the foramen. Modified Japanese Orthopaedic Association (mJOA) scores were used to assess the severity of myelopathy. These experiments were powered with effect size based on comparisons of midline and lateral stimulation in the rat (24). Conservatively assuming a reduced effect size by 50% compared with the rat experiments (Cohen’s *d* = 0.97 vs. 1.94), the analysis indicated that to achieve 90% power with a Wilcoxon signed-rank test (α = 0.05) would require 14 participants. Additional participants were recruited to map responses from each cervical segment, with a minimum of five participants from each level of the cervical enlargement (C5–T1).

**Table 1.** Clinical characteristics of participants

	Age, yr*/Sex	mJOA	Symptom Onset	Unsteady Gait	Pain	Numbness	Weakness	Hyperreflexia		Sphincter Dysfunction	Severe Foraminal Stenosis**	T2 Signal Change
								Upper Extremities	Lower Extremities			
*P01*	69/M	14	Chronic	No	Axial neck and radiating	Yes	Yes	No	No	No	C3–4, C4–C5, C7–C8	C3–C4, C4–C5
*P02*	48/F	14	Chronic	No	None	Yes	Yes	Yes	No	No	C3–C4, C4–C5, C5–C6	C2–C3, C4–C5, C5–C6
*P03*	58/M	10	Subacute	Yes	None	Yes	Yes	No	No	Yes	C4–5, C5–C6	C4–5, C5–C6
*P04*	54/M	17	Chronic	No	Radiating	Yes	No	No	No	No	C6–C7	C3–4, C6–C7
*P05*	74/F	12	Chronic	Yes	Radiating	Yes	Yes	Yes	Yes	No	C3–C4, C4–C5, C5–C6, C6–C7	C3–C7
*P06*	71/F	11	Chronic	No	Radiating	Yes	Yes	Yes	No	No	C4–C5, C6–C7	C4–5, C5–C6
*P07*	62/M	10	Chronic	Yes	None	Yes	Yes	Yes	Yes	No	C5-C6, C6–C7	C6–C7
*P08*	83/F	14	Chronic	No	Radiating	No	Yes	Yes	Yes	No	None	C3–4, C4–C5, C5–C6
*P09*	72/F	12	Subacute	No	Axial Neck	Yes	Yes	No	No	No	C4–C5, C5–C6	C3–C4
*P10*	70/F	10	Chronic	No	Radiating	Yes	Yes	Yes	No	No	None	C3–4, C4–C5, C6–C7
*P11*	74/F	13	Chronic	No	Radiating	Yes	Yes	No	No	No	C4–C5, C6–C7	C3–C4
*P12*	50/F	11	Chronic	No	Radiating	Yes	Yes	Yes	No	No	C5–C6, C6–C7, C7–C8	C5–C7
*P13*	60/M	17	Chronic	No	None	Yes	No	No	No	No	C6–C7	C3–C4
*P14*	74/M	15	Chronic	Yes	Axial neck	No	No	Yes	Yes	No	C4–C5	C4–C5
*P15*	76/M	16	Chronic	Yes	Axial neck	No	Yes	No	No	No	C3–C4, C4–C5	None
*P16*	53/M	17	Chronic	No	Radiating	Yes	No	No	No	No	None	C6–C7
*P17*	71/F	11	Chronic	Yes	Axial neck and radiating	No	Yes	Yes	Yes	No	None	C3–C4
*P18*	56/M	11	Chronic	Yes	Axial neck and radiating	No	Yes	Yes	Yes	No	C3–C4, C4–C5, C5–C6	C2–C3, C3–C4, C5–C6
*P19*	39/M	10	Chronic	Yes	None	Yes	Yes	No	No	Yes	None	C4–C5
*P20*	78/F	14	Chronic	Yes	Axial neck and upper back	No	Yes	No	No	No	C4–C5, C5–C6	C4–C5, C5–C6, C6–C7
*P21*	72/F	12	Chronic	Yes	Axial neck	No	Yes	No	No	No	C3–C4, C4–C5	C3–C4, C4–C5, C6–C7
*P22*	76/F	10	Chronic	Yes	Axial neck and radiating	Yes	Yes	No	No	Yes	None	C5–C6
*P23*	49/F	17	Chronic	Yes	Axial neck and radiating	Yes	Yes	Yes	Yes	No	None	T1–T2
*P24*	79/M	11	Chronic	Yes	None	Yes	Yes	No	Yes	No	C3–C4, C4–C5, C5–C6, C6–C7	C4–C5, C5–C6
*P25*	71/M	13	Subacute	Yes	None	Yes	Yes	No	No	No	C3–C4, C4–C5, C5–C6, C6–C7	C3–C4, C4–C5, C5–C6
*P26*	80/F	11	Chronic	Yes	Axial neck and radiating	No	Yes	Yes	No	No	C3–C4	C3–C4

All assessments were performed blinded to the electrophysiology results. *At the time of surgery. **On the stimulated side. Severe foraminal stenosis is defined as no visible cerebrospinal fluid (CSF) around the nerve root. mJOA, modified Japanese Orthopaedic Association (mJOA) score. See Supplemental Table S1 for associated experiment parameters.

### Electrophysiology

Stimulation was performed with epidural electrodes in participants undergoing clinically indicated surgery, and the muscles recorded were those chosen based on the standard montage for cervical spine surgeries performed at our institutions. After anesthesia induction, only total intravenous anesthesia was used. No anesthetic adjustments were made during the 15 min dedicated to the experiment. Recording and stimulation were performed with Cadwell Elite/PRO, Cadwell IOMAX (Cadwell Inc.), or XLTEK Protektor32 (Natus Medical Inc.) intraoperative monitoring systems. The stimulation device chosen for a particular experiment was dependent on the study site and availability (see Supplemental Table S1). The experimental procedure began once the dura was exposed and the epidural stimulation electrode was placed. Muscles were chosen for electromyogram (EMG) per standard of care (Fig. 1*A*). MEP responses were recorded with subdermal needles at a sampling rate between 6 kHz and 10.4 kHz and band-pass filtered between 10 Hz and 2,000 Hz. Epidural spinal cord stimulation was performed in trains of three pulses with a handheld double-ball tip probe (2.3-mm-diameter contacts, 10-mm spacing); single-pulse and catheter electrode stimulation were also used at specific cervical segments (see Supplemental Table S1). Stimulation with a three-pulse train was used to reduce the intensity necessary to evoke an MEP in order to reduce current spread (39). In each case, the electrodes were oriented in the rostro-caudal direction (Fig. 1*A*, *inset*) with the cathode caudal. At each testing site, stimulation was delivered every 2 s, a frequency that we determined does not alter responses with repeated stimulation (data not shown).

#### Comparison of midline vs. lateral stimulation.

Within each cervical segment, midline stimulation was compared with lateral stimulation. Midline electrode placement was determined by visual estimation and confirmed with measurement from the bony landmarks on either side. Electrodes were allowed to dimple the surface of the dura to approximately one-half the depth of the ball tip. Before the start of the experiment, we designated the lateral stimulation to be performed on the left or right to match each participant’s less impaired side based on clinical signs (see Supplemental Fig. S1), symptoms, and MRI; this was done to minimize interaction of neurological deficits on electrophysiological responses.

##### Stimulation sites and intensity.

An example of one experiment is shown in Fig. 1*C*. To begin each experiment, the stimulation electrode was placed on midline at the most caudal segment exposed during surgery. Stimulation intensity was incremented from 0 to 8 mA to assess the activation threshold and estimate the subsequent recruitment curve (minimum 5 MEPs per stimulation intensity; first panel of Fig. 1*C*). Threshold was defined as the lowest stimulation intensity to produce an MEP in the most responsive muscle at the initially tested segment. The threshold of this muscle (Supplemental Table S1) was used to set the fixed stimulus intensity (120% of threshold) for 30 s of stimulation at other segments. The experiments proceeded by repeating the stimulation intensity ramp and fixed-intensity stimulation at the equivalent lateral site (second panel of Fig. 1*C*).

#### Comparison of responses at different cervical segments.

MEPs at multiple segments were compared using the fixed intensity, which was repeated at more rostral segments (third panel onward in Fig. 1*C*). This stimulation protocol was applied in all experiments. Additional experimental conditions were tested (see Supplemental Table S1) using single-pulse stimulation and a flexible catheter electrode (1.3-mm contacts, 15-mm spacing; Ad-Tech Medical Instrument Corp). The catheter could be inserted below the lamina to allow access to segments not directly exposed surgically. We confirmed that the data from catheter stimulation produced patterns of MEPs at each segment similar to the data recorded with ball electrodes and that exclusion of these data did not substantially change the results (see Supplemental Fig. S2).

### Data Analysis

Data was exported from proprietary intraoperative monitoring software to MATLAB (R2020b) and Python (v3.8), where analysis was performed. MEPs were quantified with the rectified area under the curve (AUC) calculated in a window between 6.5 ms and 75 ms after the start of the first stimulation pulse. To indicate the absence or presence of MEPs (dashed line in Fig. 8; cutoff in Fig. 10), an equivalent estimate of the 50 µV threshold was used, as is typical in noninvasive studies (40). This value was estimated by regressing AUC onto the peak-peak MEP size, resulting in an AUC of 0.33 µVs.

#### Statistical analysis.

Values are reported as mean ± 2 × standard error of the mean (SE) except in cases where the median is used. Nonparametric statistical tests are used throughout (Wilcoxon rank-sum and signed-rank tests, α = 0.05).

#### Artifact rejection.

Rejection was based on principal component analysis and confirmation by a human observer. The principal components were computed for a specific muscle and electrode location across multiple stimulation intensities. These principal components captured the shape of the MEPs and were regressed with each MEP. The root mean square of the regression error was then used to rank the responses. This ranking sorted the responses so that the most dissimilar waveform shapes were ranked highest. A manually adjusted sliding scale was then used to reject the highest-ranked traces that did not appear physiological under visual inspection: deflections in baseline, spread of stimulation artifact into the evoked response, excessive line noise, and fluctuations that were not time-locked to other responses. This led to 1,736 of the 116,419 MEPs (1.5%) being rejected.

#### Midline-lateral comparisons.

**Figure 3.**
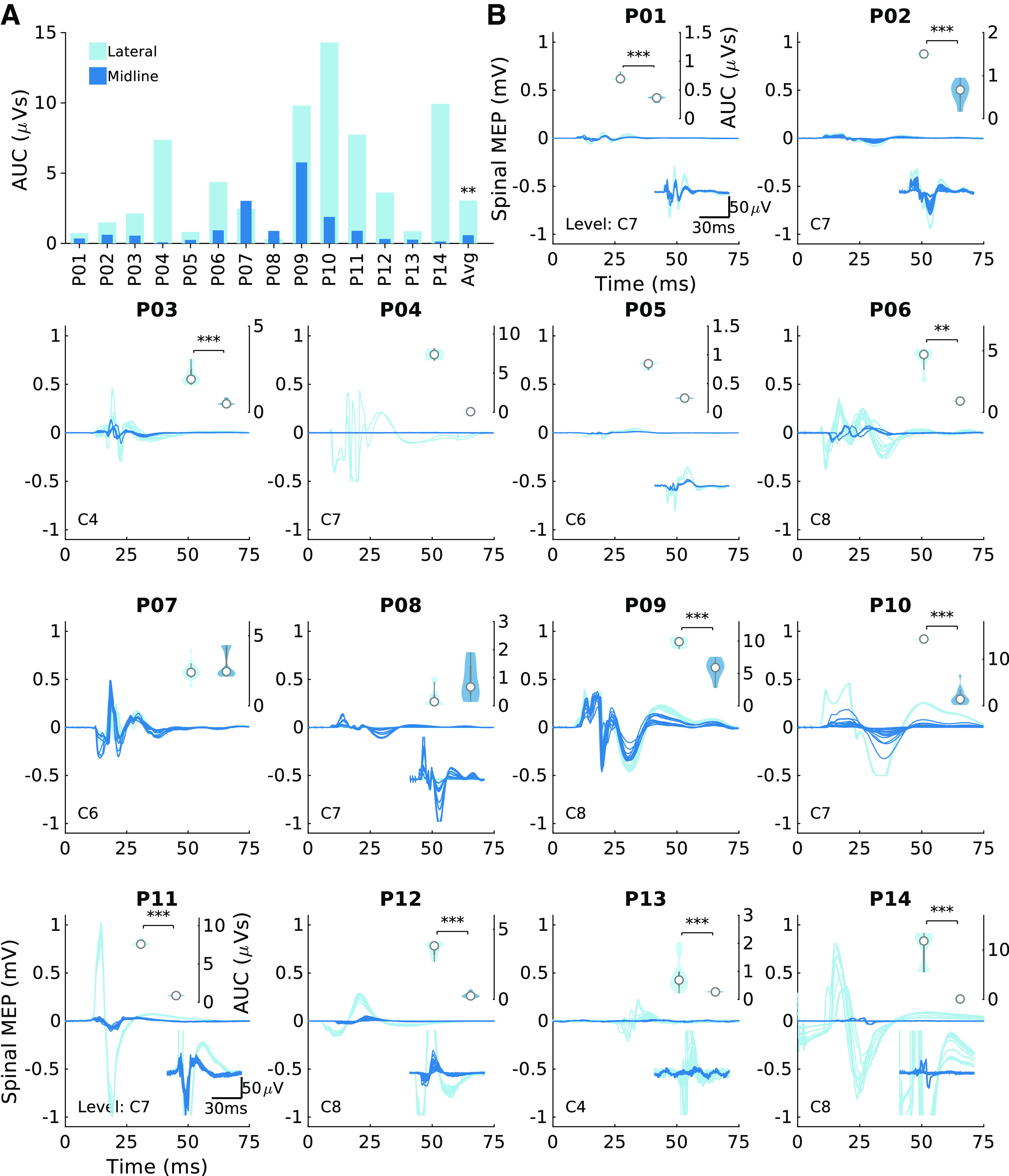
Lateral stimulation is more effective than midline stimulation. *A*: summary of the rectified area under the curve (AUC) of the triceps muscle at the most caudal segment where a response was present. Midline simulation (dark color) and lateral (light color) stimulation were performed at the fixed intensity for each participant. Lateral stimulation produced consistently larger responses for the most caudal responsive segments. A signed-rank test was applied between the midline and lateral conditions. Average (Avg) bar represents the median, **P < 0.01. *B*: individual motor evoked potentials (MEPs) that compose the summary plot for individual participants. *Bottom inset* of each panel represents a magnification of the MEP in cases in which the response is small. *Top inset* of each panel shows a violin plot of AUC (white circle represents the median). Within-participant Wilcoxon rank-sum tests were conducted in individual participants. **P < 0.01, ***P < 0.001, Bonferroni corrected for multiple comparisons; *n* = 14 participants, 6 male/8 female. Note that for *P04*, *P09*, and *P10* the signal saturated at 0.5 mV because of a limitation of the recording hardware.

**Figure 4.**
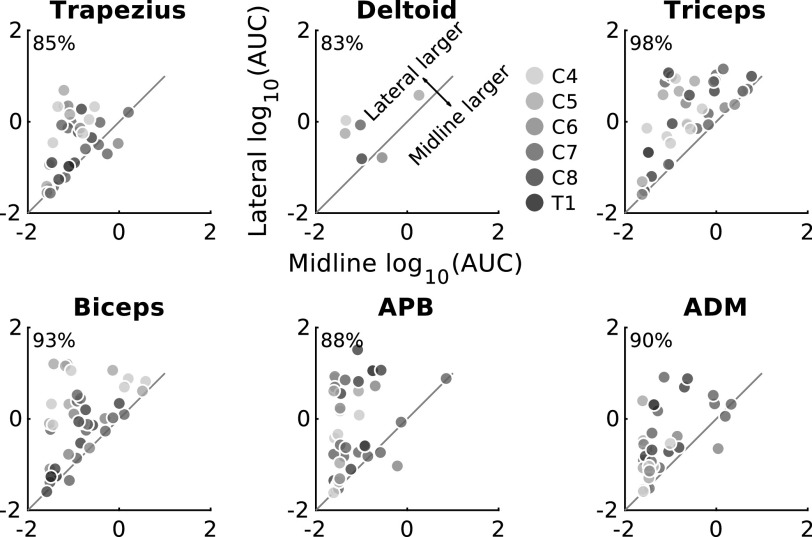
Lateral stimulation is more effective than midline stimulation regardless of stimulation segment or recorded muscle. Area under the curve (AUC) from all segments and muscles for all participants in whom both midline and lateral stimulation was performed. AUC was larger when stimulation was applied laterally than at midline in the majority of tested cases. The percentage of motor evoked potentials (MEPs) that were larger in lateral than midline stimulation is shown in the *top left* corner of each plot. *n* = 14 participants, 6 male/8 female. ADM, abductor digiti minimi; APB, abductor pollicis brevis.

**Figure 5.**
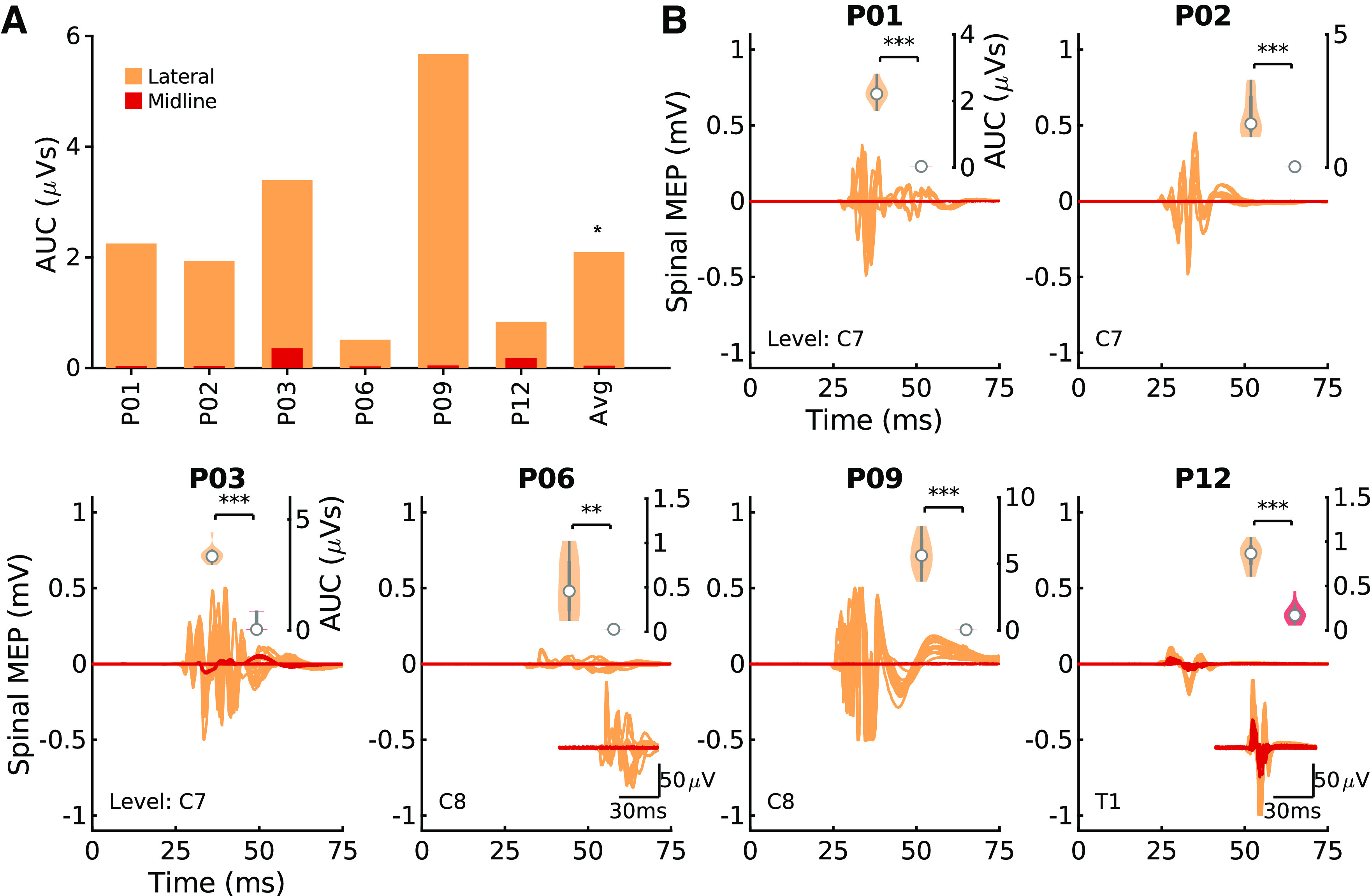
A subset of participants display activation in their leg muscles when stimulation is applied in the cervical cord. *A*: summary of the rectified area under the curve (AUC) of the tibialis anterior (TA) muscle at the most caudal segment where a response was present. Midline simulation (dark color) and lateral (light color) stimulation was performed at the fixed intensity for each participant. Motor evoked potentials (MEPs) are shown for the most caudal responsive segment. In a subset of participants that display activation of TA (AUC > 0.33 μVs), lateral stimulation produced consistently larger responses. A signed-rank test was applied between the midline and lateral conditions; the average (Avg) bar represents the median, *P < 0.05. *B*: individual spinal MEPs that compose the summary plot for individual participants. *Bottom inset* of each panel represents a magnification of the MEP in cases where the response was small. *Top inset* of each panel shows a violin plot of area under the curve (AUC) (white circle represents the median). Within-participant Wilcoxon rank-sum tests were conducted in individual participants. *P < 0.05, **P < 0.01, ***P < 0.001, Bonferroni corrected for multiple comparisons; *n* = 6 participants, 2 male/4 female. Note that for *P09* the signal saturated at 0.5 mV because of a limitation of the recording hardware.

The stimulation efficacy of midline stimulation was compared to lateral stimulation in four ways. First, the MEPs for midline and lateral stimulation at 120% of midline threshold were compared for each individual participant (Fig. 3, *insets*), using the Wilcoxon rank-sum test, Bonferroni corrected for the number of participants (*n* = 14, all participants with triple-pulse stimulation at midline and lateral locations). Second, the mean AUC for midline and lateral stimulation across the 14 participants was compared with the Wilcoxon signed-rank test (Fig. 3*A*). The same method applied to the triceps muscle MEPs (Fig. 3) was applied to the tibialis anterior (TA) responses in Fig. 5. Third, to ensure that differences observed in specific muscles at specific segments of stimulation were general trends, all of the responses of the six arm and hand muscles were plotted in Fig. 4. The percentage of responses that were larger for lateral stimulation was computed (shown for each muscle in Fig. 4). The fourth comparison of midline versus lateral stimulation was performed with recruitment curves. The curves were used to estimate thresholds for MEPs and the rate of change of MEPs with stimulation intensity (slope). A function that approximates a linear recruitment curve was used (see *Estimation of threshold and slope section*). In a single participant in whom epidural and subdural stimulation sites were tested, midline and lateral conditions were compared with recruitment curve thresholds (Fig. 7).

#### Comparison of responses at different cervical segments.

All MEPs where the fixed-intensity stimulation was used within a single participant were included in this analysis. The average AUC across participants was computed by using the geometric mean (Fig. 8*A*). The similarity in MEPs across segments was measured with the Spearman correlation coefficient, calculated between all segments across muscles for each participant (see Fig. 8, *B1* and *B2*) and then averaged across participants (see Fig. 8*B3*). To demonstrate similarity across segments, hierarchical agglomerative clustering was applied (41). This procedure recursively calculates the correlation between conditions and merges them to create a correlation between clusters that is displayed as a dendrogram. The similarity in MEPs across segments was quantified with the Spearman correlation coefficient, calculated between all segments across muscles for each participant and then averaged across participants. The merging procedure is based on the linkage metric, which was conservatively set to the minimum distance (1 − correlation). We set the cluster cutoff at 70% of the maximum distance.

#### Midline-lateral vs. rostral-caudal and anterior-posterior comparison of waveform shape and latency.

To test the hypothesis that midline and lateral stimulation activate largely overlapping circuitry, an analysis of midline and lateral waveform shape (42) and onset latency was performed. Stimulation intensity impacts MEP size, which is known to impact MEP shape (43). Consequently, we matched MEPs according to their AUC across conditions before calculating similarity. For muscles of the arm and hand in all participants at the most caudal stimulated level, the fixed-intensity MEP size at midline was matched to the MEP size in the lateral recruitment curve at the same segment. We defined a match as an AUC difference < 0.5 µVs. If a match was found, two computations were performed to determine the similarity of the waveforms. First, the onset latency of these waveforms was computed based on the averages at each site (first deflection greater than 10 µV). Second, the Pearson correlation coefficient of these waveforms was computed; to control for differences in latency, the highest correlation between waves at different latencies (from −12.5 ms to 12.5 ms) was computed, and the highest value was used.

In contrast to midline-lateral stimulation, we expected stimulation across different rostral-caudal segments to activate different circuits and consequently produce larger differences in onset latencies and waveform shapes. To calculate these metrics across segments, the same method developed to compare midline-lateral metrics was used to compare a fixed-intensity MEP size at a rostral level to a size-matched MEP at a more caudal level, where a recruitment curve was recorded. Waveform similarities and differences in onset latencies were compared between the midline-lateral condition and the rostral-caudal condition pooled across the segments. Because of the relatively low number of matches in MEP size, individual muscles were treated as independent from each other for this analysis (as indicated by Fig. 9*A*, *insets*).

Finally, we tested the difference in responses from anterior and posterior spinal cord stimulation. This was done for two reasons: to determine whether responses are mediated by different pathways and to show that differences in responses would be detected by the waveform analysis. We expected that MEPs generated from lateral posterior stimulation would produce large differences in waveform shape compared with anterior stimulation. This analysis was possible in a single participant in whom anterior and posterior segments were exposed and the dorsal root entry zone and corresponding ventral root location could be targeted. To determine whether waveform shape was more or less different in the anterior-posterior condition compared with the midline-lateral condition, the anterior-posterior similarity metric was ranked relative to the midline-lateral metrics and reported as a percentile. The latency difference was not calculated for this condition because stimulation artifacts caused baseline shifts that made the onset time estimation unreliable.

#### Estimating the change in MEP produced by moving electrodes midline to lateral vs. between segments.

Comparisons between movement of electrodes along both the midline-lateral and rostro-caudal axes was possible in nine participants. The segment of maximum AUC caused by lateral stimulation was first identified for each muscle group; subsequently, the differences between MEP sizes at this and neighboring segment(s) were calculated. If two neighboring segments were present, the two differences were calculated and then averaged. Similarly, the difference between the lateral MEP and midline MEP sizes at the same segment was calculated for each muscle. The differences in MEPs were then normalized by distance by dividing MEP values by a generic interroot length for the rostro-caudal axis and half the transverse cord diameter for the midline versus lateral placement. Distance estimates were derived from a human cadaveric study (44), in which interroot distances in the cervical spinal cord (average 12.5 mm) and midline- to lateral distance (average 6.7 mm) were measured. Each participant had the MEP sizes across muscles averaged; a Wilcoxon signed-rank test was applied to determine the relative change along each axis.

#### Estimation of threshold and slope.

Recruitment curves were collected at only one segment per participant because of time constraints. Recruitment curves were fit to enable comparison of slope and threshold across multiple segments. Specifically, the stimulation intensity and AUC relationship was modeled as a softplus function: $y_{j,m,l}=o_{j,m}+\frac{b_{j,m,l}}{k}log\left(1+ke^{x-x_{j,m,l}^{\mathrm{th}}}\right)$, where *y_j_*_,_*_m_*_,_*_l_* represents the AUC for a given participant (*j*), muscle (*m*), and electrode location (*l*) for a given stimulation intensity (*x*). Symbols *o*, *b*, and *x*^th^ represent the recruitment curve offset, slope, and threshold, respectively. The parameter *k* was set to 20 to approximate a linear rectified function while maintaining numerical stability. A fit was performed with a generalized pattern search algorithm (see Supplemental Methods) for every muscle and participant simultaneously for multiple locations on the spinal cord (because of the shared offset). A shared offset was used because a subset of data did not test a stimulus intensity below MEP threshold. To compare thresholds across the midline and lateral conditions across participants, a Wilcoxon signed-rank test was used on the difference between midline and lateral estimates averaged across segments and muscles. The same procedure was used for a comparison of the slope estimates.

## RESULTS

### Participant Recruitment and Characteristics

Participants (*n* = 26, 12 male and 14 female) underwent surgery that exposed the cervical enlargement for electrical stimulation; their demographics and clinical findings are summarized in Table 1. Mean age of study participants was 66 yr (range 39–83). Twenty-three patients had chronic symptoms, and three patients had subacute symptoms.

### Location of Stimulating Electrodes

The location of the stimulating electrodes was determined in two ways. First, electrode location based on anatomical landmarks (Fig. 2*A1*) was identified in an intraoperative CT (Fig. 2*A2*) and then coregistered (Fig. 2*A3*) into MRI space in one participant. The location of the electrode was confirmed to be over the C7 dorsal root entry (Fig. 2*A4*), where it had been placed intraoperatively.

As a secondary confirmation of the electrode placement strategy, an additional experiment was performed in a patient undergoing laminectomy and dural opening for resection of intradural meningioma. Electrodes were placed relative to anatomical landmarks as for epidural stimulation, and once the dura was removed confirmation was made that the location matched the dorsal root entry zone (Fig. 2*B*).

### Lateral Stimulation Is More Effective than Midline Stimulation

Comparison of spinal motor evoked potentials (MEPs) to stimulation at midline and lateral locations was performed in 14 participants. We confirmed that placing the electrodes in the lateral position located them over the dorsal root entry zone, where afferent axons enter the spinal cord (Fig. 2). Figure 3 shows MEPs in response to midline (dark) or lateral (light) stimulation, using an intensity set to 120% of the threshold for midline MEPs. Lateral stimulation of the cervical spinal cord generated larger MEPs than midline stimulation across participants (Fig. 3*A*; mean midline AUC = 1.14 ± 0.82 µVs, lateral AUC = 4.71 ± 2.28 µVs; Wilcoxon signed-rank test, *P* = 0.002, *n* = 14). These changes correspond to a median increase of 258% in the MEP when stimulating laterally versus at midline. The subplots in Fig. 3*B* show raw MEPs of the 14 participants. The top insets show violin plots of the lateral and midline MEPs for each individual, with asterisks indicating significant differences (*P* < 0.05, Bonferroni-corrected Wilcoxon rank-sum test). Ten of 14 participants showed significantly increased MEPs with lateral stimulation compared with midline.

The greater effectiveness of lateral compared to midline stimulation was not unique to a specific muscle or any particular segment. Figure 4 shows each of the six arm muscles in which MEPs were recorded and the six spinal segments that were stimulated. Each dot represents the lateral (*y*-axis) and midline (*x*-axis) responses to stimulation at the various segments in each of the participants. All responses above the *x* = *y* line represent larger lateral than midline MEPs. On average, across participants, segments, and muscles, 91.5% of MEPs were larger when the spinal cord was stimulated laterally.

The increased effectiveness of lateral stimulation in the cervical cord was also observed in the leg muscles. Figure 5*A* shows the summary of responses for midline (dark) or lateral (light) stimulation. In the 6 out of 14 participants who had tibialis anterior (TA) MEPs in response to cervical stimulation, lateral cervical stimulation generated larger leg MEPs than midline stimulation (AUC = 0.11 ± 0.10 µVs, lateral AUC = 2.43 ± 1.54 µVs; Wilcoxon signed-rank test, *P* = 0.031, *n* = 6). The subplots in Fig. 5 show raw MEPs of the six participants. The top insets show a violin plot of the lateral and midline MEPs for that individual (*P* < 0.05, Bonferroni-corrected Wilcoxon rank-sum test). All of the six participants showed significantly increased MEPs with lateral stimulation compared with midline.

### Lateral MEPs Are Larger because of Lower Thresholds and Steeper Recruitment Curves

**Figure 6.**
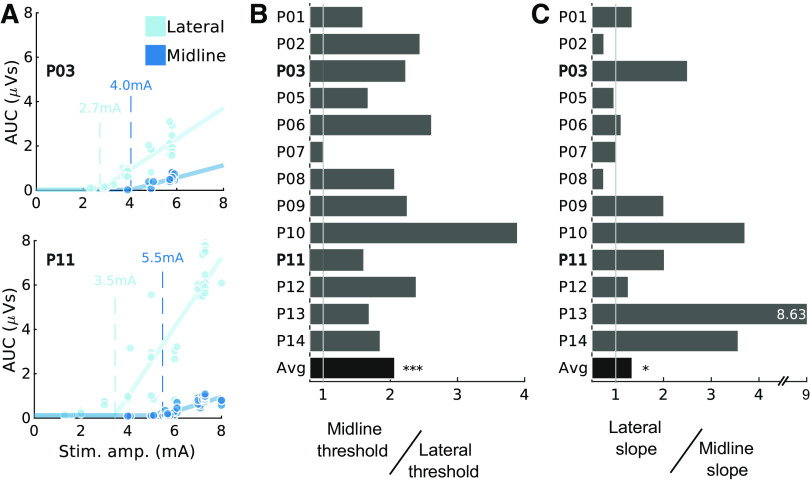
Larger lateral motor evoked potentials (MEPs) are driven by both a reduction in threshold and increase in slope. *A*: the threshold of muscle activation was determined by fitting functions to sections of data where multiple stimulation intensities were used. Circles indicate individual data points (light color, lateral; dark color, midline), solid lines show example fitted functions, and dashed lines show estimates of threshold. Triceps muscle is shown for 2 participants as indicated. *B*: the relative efficacy of lateral stimulation can be summarized as midline threshold ÷ lateral threshold (lower lateral threshold indicates higher lateral efficacy). The majority of participants showed lower lateral than midline threshold. *n* = 13 participants, 5 male (M)/8 female (F), Wilcoxon signed-rank test, ***P < 0.001. *C*: lateral to midline slope ratio (lateral slope ÷ midline slope). The majority of participants showed steeper recruitment slopes from stimulation at lateral sites. *n* = 13 participants, 5 M/8F, Wilcoxon signed-rank test, *P < 0.05. Note that 1 participant has been omitted (*P04*) as there was insufficient range in tested stimulation intensities to perform model fitting. Average (Avg) bar represents the median. AUC, area under the curve.

The increased effectiveness of lateral stimulation may be driven by a reduction in the threshold for recruitment, an increase in the rate of change of MEP size with stimulation intensity (slope of the recruitment), or both. Recruitment curves were fitted to estimate these parameters directly from data where multiple stimulation intensities were tested at the same segment (e.g., Fig. 1*C*, C7). Examples of the recruitment curve threshold and slope estimation procedure for the triceps muscle of two participants are shown in Fig. 6*A* for midline and lateral stimulation. Across participants, average threshold increased 107% for midline stimulation over lateral stimulation, indicating that higher stimulus intensity was needed to evoke an MEP (*P* = 2 × 10^−4^, Wilcoxon signed-rank test, *n* = 13; Fig. 6*B*). The rate of increase of MEP size with stimulation intensity was also influenced by the site of stimulation, with a median increase in the slope of 34% for lateral stimulation compared to midline stimulation (*P* = 0.010, Wilcoxon signed-rank test, *n* = 13; Fig. 6*C*). Thus, lateral stimulation was more effective than midline stimulation both because of a lower threshold and because of an increased slope of the recruitment curve.

### Lateral Stimulation Efficacy Is Most Pronounced When Applied under the Dura

**Figure 7.**
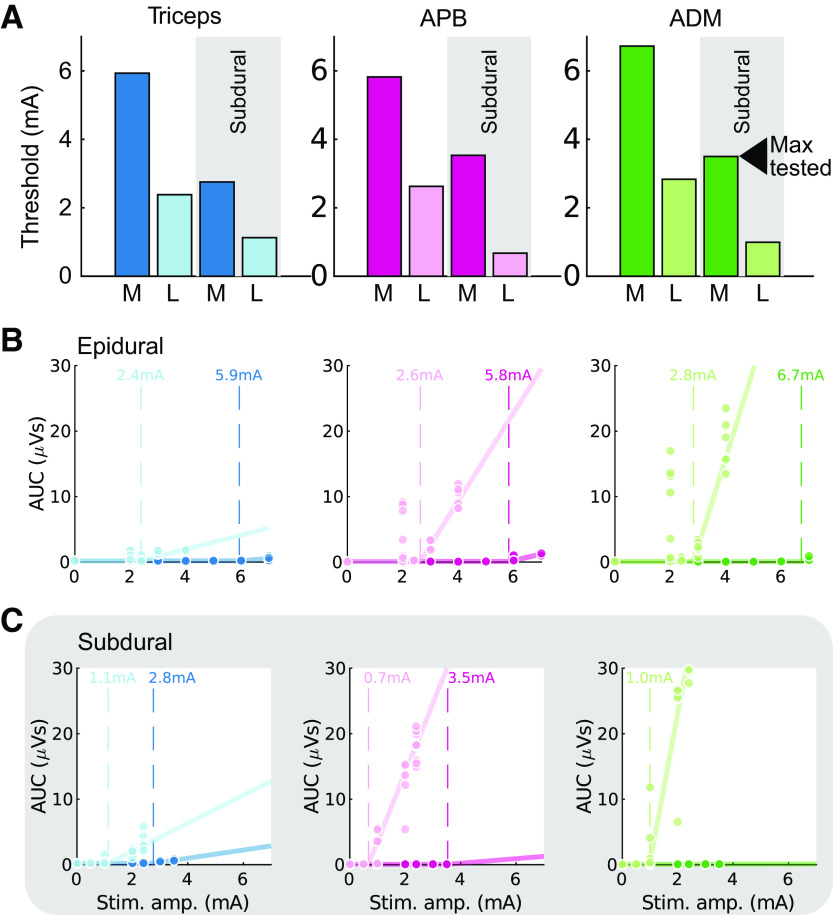
Comparison of subdural and epidural stimulation intensity thresholds at midline and laterally. *A*: summary of thresholds for midline (M, dark colors) and lateral (L, light colors) epidural and subdural stimulation in a single participant (*P23*, female). Lateral subdural stimulation is most effective, followed by lateral epidural stimulation, midline subdural stimulation, and midline epidural stimulation across the 3 activated muscles. Electrode position is shown in Fig. 2*B*. ADM, abductor digiti minimi; APB, abductor pollicis brevis. *B*: the threshold of muscle activation for epidural stimulation was determined by fitting functions to sections of data where multiple stimulation intensities were used. Circles indicate individual data points, solid lines show fitted functions, and dashed lines show estimates of threshold. AUC, area under the curve. *C*: as for *B*, with data from the corresponding subdural stimulation location.

Stimulation was applied midline and laterally below the dura at the T1 root level and at matched epidural locations. Across the three muscles activated (Fig. 7, *A* and *B*), the lowest intensity stimulation was found for stimulation of the dorsal root entry zone below the surface of the dura, followed by lateral epidural, midline subdural, and finally midline epidural stimulation locations.

### Cervical Dorsal Stimulation Evokes MEPs in Muscles Innervated at That Segment but Also Muscles Innervated at Adjacent and Remote Segments

**Figure 8.**
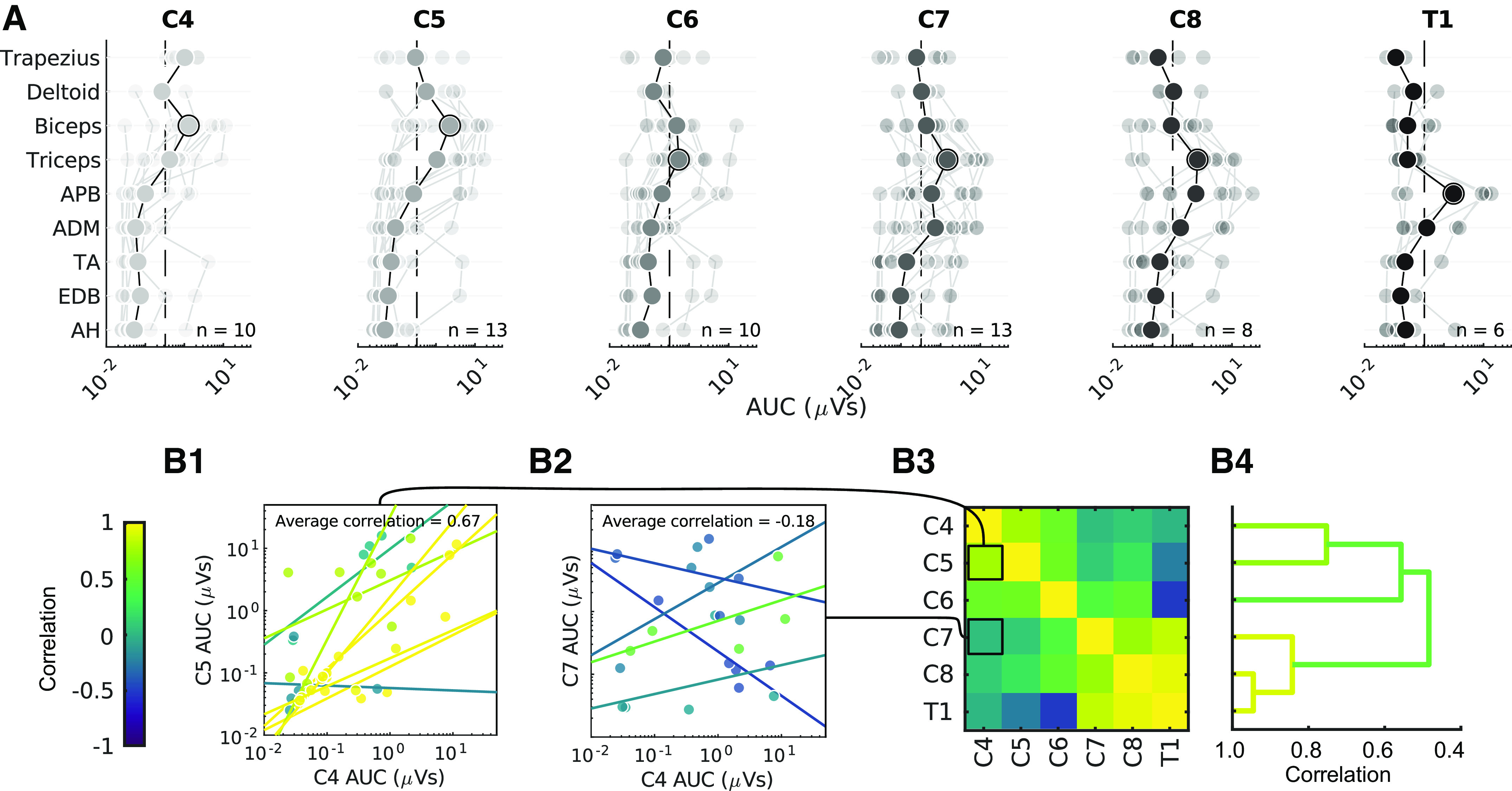
Rostral-caudal distribution of motor evoked potentials (MEPs). *A*: MEPs for each of the recorded muscles with stimulation are shown for lateral stimulation at segments C4–T1. The data are plotted on a log scale because of the exponential nature of MEPs to show their relative size. The average is shown in bold. Faint lines represent individual participant data. *n* = 26 participants, 12 male/14 female, with the number of participants represented shown within each panel. Dashed line (0.33 µVs) indicates a threshold for presence of MEPs (see materials and methods). ADM, abductor digiti minimi; AH, abductor hallucis; APB, abductor pollicis brevis; EDB, extensor digitorum brevis; TA, tibialis anterior. Equivalent figure with T2 signal change segments excluded is shown in Supplemental Fig. S1. *B*: within-participant similarity (correlation) of muscle responses at different segments, averaged across participants. *B1* and *B2*: example area under the curve (AUC) data correlations when comparing C4 to C5 (*B1*) or to a more distant C7 (*B2*). Nearby segments are highly correlated for the majority of participants (colors represent different participants), whereas more distant segments are not. *B3*: muscle activation appears to form a distinct cluster formed from lower cervical segments (C7, C8, T1). *B4*: dendrogram constructed from hierarchical clustering of *B3*. Individual pairs of correlations are merged into clusters, and the maximum correlation between the entries in these clusters is represented by the width of the merge. Colors represent the maximum correlation within distinct clusters (see materials and methods).

Although we predicted a sharp difference in responses between midline and lateral stimulation, we expected smaller differences with movement between cervical segments along the rostro-caudal axis (Fig. 1*B*). MEPs were recorded in arm and leg muscles after a fixed-intensity stimulation at six different spinal segments (C4–T1). Figure 8*A* shows MEP values for each muscle with stimulation at the segments available for a particular participant. Individual participants are represented by faint lines and the group average for each muscle by bold lines. The dashed lines indicate a threshold for the presence of MEPs. The largest MEPs were observed in the muscles innervated at the stimulated segment. For example, biceps activation was dominant at C4 and C5, triceps at C6, C7, and C8, and abductor pollicis brevis (APB) at T1. Triceps MEPs were present at all segments except for T1. Additionally, the MEP latencies show an increase from arm, to hand, to leg (Supplemental Fig. S3). As shown in Fig. 5, some participants had MEPs in the legs, although those were always smaller.

The patterns of responses are likely to reveal how similar each segment is to the other segments. To determine this, we calculated the correlation between segments of MEPs from all muscles. Figure 8, *B1* and *B2*, show the results of all the individual participants who had stimulation at the two segments indicated on the axes. Each dot corresponds to the MEP for an individual muscle, and all the arm and hand muscles for an individual are used to create a linear correlation represented by a line. By averaging these correlations across participants (Fig. 8*B3*), we were able to examine the similarity of muscle responses across segments without the confound of across-participant variability. MEPs from C7–T1 largely fell into a cluster more distinct from rostral levels. We used a clustering algorithm to show these relationships; the correlation within the C7–T1 cluster averaged 0.85, whereas the correlation within the C4–C6 cluster averaged 0.57. The correlation between the rostral and caudal clusters was 0.5. Thus, the caudal cluster is strongly related internally, with much lower correlations to other levels.

Our goal was to understand the organization of the intact cervical spinal cord, but the study was performed in people with myelopathy. To facilitate the greatest responses, we performed lateral stimulation on the less affected side. In addition, we performed an analysis similar to Fig. 8 but excluding responses in all segments where T2 signal change was present and separately excluding responses in all segments where severe foraminal stenosis was present on the stimulated side. Exclusion of the sites did not change the pattern of responses significantly (average correlation between MEP size with and without T2 signal change segments excluded *r* = 0.88, without several foraminal stenosis segments *r* = 0.92; Supplemental Figs. S4 and S5). This high correlation suggests that the patterns of muscle activation from cervical spinal cord stimulation were driven largely by the activation of intrinsic circuits that were spared from the effects of myelopathy.

### Similar Latencies and Waveforms from Midline and Lateral Stimulation Matched for Size

**Figure 9.**
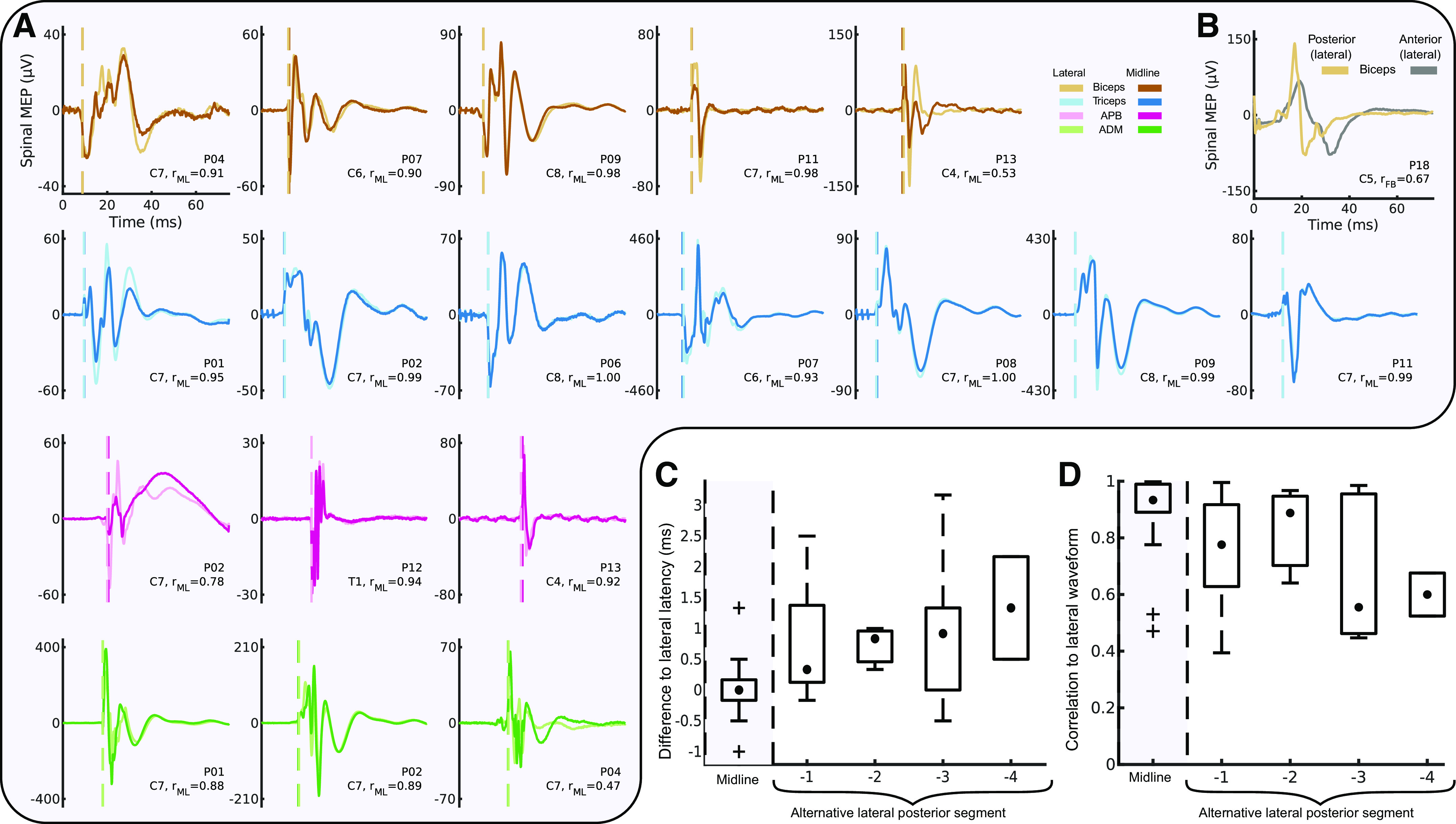
Comparison of midline-lateral, rostral-caudal, and posterior-anterior waveform shapes. *A*: averaged midline (dark colors) and lateral (light colors) motor evoked potentials (MEPs) matched for size are highly similar. *r*_ML_ indicates the correlation between the 2 waveforms after adjusting for potential differences in onset latencies. ADM, abductor digiti minimi; APB, abductor pollicis brevis. *B*: waveform shapes for posterior-anterior comparison for a single participant (*P18*) in the biceps muscle appear highly distinct from each other. *C*: box plot of onset latencies for midline and lateral MEPs as in *A* (gray background), compared to onset latencies between lateral MEPs and MEPs at alternative lateral segments (white background). Midline-lateral latency differences are centered around 0 ms, whereas latency differences increase with more distant segments. *n* = 12 participants, 4 male (M)/8 female (F); note that data are pooled across muscles. *D*: box plot of the averaged midline and lateral MEPs as in *A* (gray background), compared to correlations between lateral MEPs and MEPs at alternative lateral segments (white background). Midline-lateral correlations are higher than neighboring rostral-caudal correlations, which appear to decrease at more distant segments. *n* = 13 participants, 5 M/8F; note that data are pooled across muscles. Hinges represent 1st and 3rd quartile, and whiskers span the range of the data not considered outliers [defined as q_3_ + 1.5 × (q_3_ − q_1_) or less than q_1_ − 1.5 × (q_3_ − q_1_)].

We expect that both midline and lateral stimulation recruit large-diameter afferent axons as they enter the spinal cord. Although lateral stimulation was far more effective at producing MEPs, we expected that the latency and waveform of the resultant MEPs from stimulation in each location would be similar when matched for the size of the MEP (see materials and methods). We examined this by comparing variation in latencies and waveform shape correlations along the midline-lateral (Fig. 9*A*) and rostral-caudal axes. For the single participant who underwent anterior and posterior surgery, the waveform shapes of the biceps MEPs from the two stimulation aspects appear highly distinct (Fig. 9*B*), and this is reflected in a low correlation (*r* = 0.67, 11th percentile of the posterior midline and lateral MEP correlations). The difference in onset latencies between midline and lateral responses was nearly identical (0.04 ± 0.22 ms, *n* = 18), and this was not different from 0 ms (*P* = 0.655, *n* = 18). In contrast, the latency from lateral responses at various levels was different (Fig. 9*C*), with longer latencies from progressively more distant segments; pooled across segments, the latency was different from the midline-lateral condition (0.83 ± 0.22 ms, *n* = 24, *P* = 0.002). We also tested the similarity in waveforms between conditions. MEP waveforms were very similar between midline and lateral stimulation (*r* = 0.89 ± 0.04, *n* = 18; Fig. 9*D*). The similarity became less across more distant segments (Fig. 9*D*); waveforms were more similar for the midline-lateral correlation than across segments (pooled *r* = 0.72 ± 0.04, *n* = 24, *P* = 0.006).

### Map of MEPs Produced by Epidural Stimulation of the Cervical Spinal Cord

**Figure 10.**
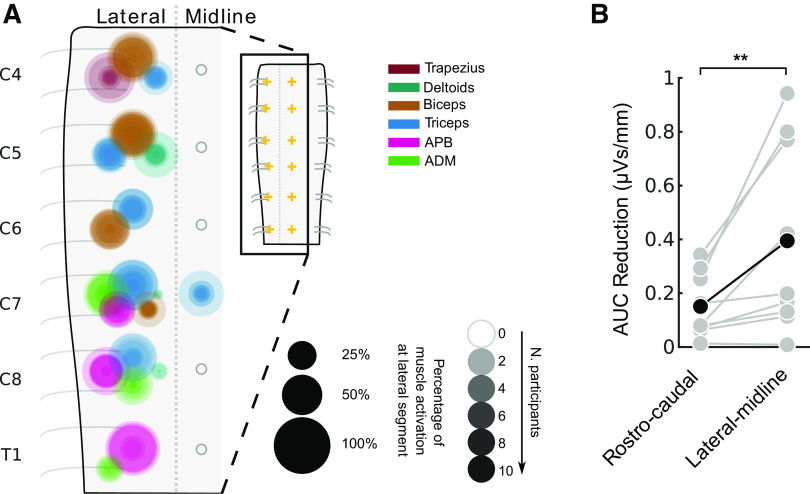
An actionable map for epidural stimulation of the cervical spinal cord. *A*: area of overlaid circles represents the rectified area under the curve (AUC) of individual participants. Activation is only drawn when the across-participant average AUC is >0.33 µVs (see materials and methods). Empty circles indicate that no muscle activation reached this threshold. Data shown here are a summarized representation of data shown in Fig. 8*A*. *n* = 26 participants, 12 male/14 female. ADM, abductor digiti minimi; APB, abductor pollicis brevis. *Inset*: crosses mark locations of stimulation. *B*: greater change in AUC in the lateral-midline axis compared to the rostro-caudal axis, *n* = 9 participants, 7 male/2 female, Wilcoxon signed-rank test, **P < 0.01. Individuals are shown as gray dots with lines connecting their values and the average of the participants in black.

Figure 10*A* summarizes the muscles activated by stimulation at each cervical segment. Circle area corresponds to the percentage of total MEP size contributed by specific muscles (designated by color), and the darkness of the circle corresponds to the number of participants. Only MEPs that were on average >0.33 µVs are shown, highlighting the dominant muscles at each segment. Although all lateral stimulation sites met this threshold, the only midline response above this threshold was the triceps MEP at C7. This map demonstrates the activation of specific muscles at each segment of the cervical spinal cord.

Differences between MEP size were larger between midline and lateral stimulation within segments than between segments. To quantify this, we determined change in MEPs as a function of distance: how much does the MEP change with each millimeter that the stimulating electrode was moved in each direction? The average MEP size was reduced at a rate of 0.39 ± 0.24 µVs/mm when moving from lateral to midline. On the other hand, MEP size from stimulation of neighboring segments was reduced at a lower rate of 0.15 ± 0.08 µVs/mm. The rate of change was more than double for lateral-midline electrode position compared with rostro-caudal position, and this difference was highly significant (*P* = 0.008, *n* = 9).

## DISCUSSION

In addition to the empiric map, these results demonstrate the underlying organization of the human cervical spinal cord. The largest changes in stimulus response were observed between midline and lateral locations, which is likely due to the greater efficacy of recruitment when the electrodes were placed over the dorsal root entry zone. Consistent with this, the relative efficacy of lateral stimulation was maintained when stimulating below the dura over the dorsal root entry zone. Movement of stimulating electrodes along the rostro-caudal axis resulted in more subtle differences in motor responses, suggesting the presence of strong overlap with adjoining segments. Clustering of these responses was particularly strong within lower cervical segments (C7, C8, T1), with more diversity at other levels. Finally, we observed MEPs arising from distant segments of the cervical spinal cord and from leg muscles, indicating potential activation of propriospinal connections (16, 45). Thus, the summary map of responses (Fig. 10*A*) provides a guide to targeting muscles via dorsal cervical spinal cord stimulation but also an organizational map of neural connections that can be recruited with this technique.

The observed differences in MEP size between midline and lateral stimulation and between neighboring segments have support in the literature, but direct evidence has been limited. The majority of work suggesting that the afferent fibers entering the dorsal root entry zone are the most sensitive neural structures to epidural stimulation comes from the lumbar cord (46). However, differences in anatomy (38) and circuitry (16) mean that findings in the lumbar cord do not directly translate to findings in the cervical cord. Recent work in simulations of current flow in monkeys and in five human participants (23) suggests that the primary mechanism is indeed the same as for the lumbar cord. However, Telkes et al. (47) compared midline and lateral stimulation for activation of leg muscles and found that some were most efficiently activated along the midline. This suggests that the optimal midline-lateral position for stimulation has not been fully resolved between the various spinal segments or other parameters.

Additionally, our work provides novel electrophysiological evidence that midline and lateral stimulation predominantly activate the same circuitry, whereas stimulation at more distant lateral rostral-caudal sites incorporates additionally more diverse circuits. The significantly different MEP morphology in response to anterior stimulation suggests that the MEPs generated in response to dorsal stimulation are indeed mediated through the dorsal afferent pathways rather than through direct activation of the motor neuron via spread to the ventral horn. This is corroborated by the subdural replication of efficacy of lateral stimulation relative to midline stimulation. Interestingly, the effectiveness of subdural stimulation relative to epidural stimulation was larger than expected based on work in monkeys (25), perhaps because of increased shunting of current from a larger volume of cerebrospinal fluid.

Unexpected spread of responses beyond the stimulated segment has been reported (33), even with intraspinal cervical stimulation (39, 48). Activation of leg muscles from stimulation of the cervical cord has also been found (33–35, 49). Consistent with these findings, we observed long-range activation, for example triceps activation with C4 stimulation and leg activation with cervical stimulation. Such activation cannot be explained by known locations of motor pools and intermingling of motoneuron cell bodies (26, 27) and suggests the presence of long-distance connections within the cervical enlargement (29, 50, 51) and between the cervical and lumbar enlargements (16, 52–55). Importantly, we show that lateral stimulation is more effective than midline stimulation for these long-distance connections.

Clustering of lower cervical responses may reflect the intrinsic organization of the spinal cord in humans. In the monkey cervical cord with intraspinal microstimulation, Moritz et al. (39) demonstrated some distinct, yet overlapping grouping of synergists such as wrist extensors and wrist flexors. They also observed considerable coactivation of agonist-antagonist pairs such as biceps and triceps. Greiner et al. (23) showed separation of biceps and triceps by comparing MEPs from stimulation at the C6–C8 segments of monkeys. However, our results suggest that the distinction between biceps and triceps muscle activation may be less pronounced because of the widespread activation of the triceps muscle. Rather, our results suggest a division between the muscles of the hand and muscles of the arm. This organization could be engaged to foster adaptive patterns of arm and hand muscle activation.

The patterns of muscle activation from dorsal spinal cord stimulation can be provisionally inferred through known spinal circuits. Spread between adjacent segments (for example, strong activation of biceps from stimulation at C4) may be mediated by the spread of afferent connections into the spinal cord or the spread of longitudinally extensive motor pools out of the spinal cord. Some observed responses may be partially driven by nonbiological spread of the electric field across levels (21), but the presence of leg muscle MEPs provides strong evidence that spinal cord pathways represent a critical mechanism. Additionally, we observe a much larger change in MEP size when electrodes are moved in the midline-lateral orientation than when they are moved in the rostral-caudal orientation, suggestive of the limitations of current spread.

Although the operating room provides a unique experimental setting, it may also limit the generalizability of our findings. Participants had myelopathy and/or radiculopathy, and either or both of these pathologies may affect axonal conduction, neuronal signaling, or synaptic connectivity. We did, however, perform a sensitivity analysis to attempt to distinguish these effects. Excluding data from the segments with T2 hyperintensity, indicative of myelopathy, did not change the overall structure of the activation map. Likewise, excluding data from segments with severe foraminal stenosis on the stimulated side, indicative of radiculopathy, did not change the overall structure of the activation map.

In addition, MEP size may also have been affected by general anesthesia and position. However, all participants were maintained on total intravenous anesthesia, consistent with standard operative and intraoperative neuromonitoring conditions; in all our experiments we observed clear, reproducible responses at multiple tested segments. Importantly, anesthetic conditions had reached a steady state during recording, and no changes were made during the experiment. Because participants were tested in an immobilized position on the operating table, we were not able to address how epidural stimulation can be used to generate movement (23, 49, 56) or how MEPs interact with body position (57–60).

Patient safety limits and time constraints did not allow us to test MEPs to their maximum value. Normalization of MEPs by their maximal value is indicative of the proportion of recruited motoneurons and less impacted by sources of variability such as muscle size and electrode position. When within-condition normalization of an individual muscle’s MEP is not possible because of the absence of saturation, some authors (15, 23) have addressed this by normalization to the maximum MEP across all tested conditions. However, this method is reliant on evidence that MEPs can be consistently driven to saturation. We have observed saturation of MEPs in only 3.4% of tested conditions, and data have consequently been presented without normalization. Although the participant number is sufficient to produce a response map consistent with the known biology, this approach may have led to some systematic over- or underrepresentation of specific muscles.

Testing during surgery also limited the muscles recorded and parameters tested. Muscle selection is chosen based on the cervical nerve roots that are most at risk of iatrogenic injury. Spinal cord function can be adequately monitored with a minimal amount of upper and lower extremity recording sites, but multiple muscle groups are required to be nerve root specific. Because wrist muscles were omitted, the observed rostral-caudal clustering of muscle activation is likely to be only partially reflective of the clustering of the circuitry of the spinal cord. Stimulation technique or study population may also be critical for the specific form of the observed clustering. Although the stronger lateral than midline stimulation effect is ubiquitous within the range of parameters that we have tested, it is possible that it may not generalize to different stimulation frequencies (2) or electrode locations. In future studies these limitations may be addressed in alternative experimental settings either noninvasively or with implanted leads.

The map of muscle responses to cervical epidural stimulation can facilitate better understanding of spinal circuits and help target interventions, which have historically been focused on activation of circuitry underlying the midline. Stimulation of the cervical spinal cord may reveal patterns of activation, whether mediated by motor primitives or complex movements. In addition to single or short trains of stimuli, longer trains and/or multisite stimulation may help to reveal these motor programs. Multiple sites of stimulation may be needed to activate the desired circuit activation or movement. This map may be used to guide further experiments to elucidate optimal sites and stimulation patterns for activating movement and strengthening damaged spinal circuits.

## DATA AVAILABILITY

The data that support the findings of this study are available from the corresponding authors, upon reasonable request.

## SUPPLEMENTAL MATERIAL

10.6084/m9.figshare.19891966 Supplemental materials: https://doi.org/10.6084/m9.figshare.19891966.

## GRANTS

Research reported in this publication was supported by the National Institute of Neurological Disorders and Stroke of the National Institutes of Health under award number 1R01NS124224 and by the Travis Roy Foundation, Boston, MA (Investigator Initiated).

## DISCLOSURES

J. B. Carmel is a founder and stockholder in BackStop Neural and a scientific advisor for SharperSense. M. S. Virk has been a consultant and has received honorarium from Depuy Synthes, Nuvasive, and BrainLab Inc; he is on the Medical Advisory Board and owns stock with OnPoint Surgical. N. Y. Harel is a consultant for RubiconMD. K. D. Riew: consulting: Happe Spine (nonfinancial), Nuvasive; royalties: Biomet; speaking and/or teaching arrangements: Biomet, Medtronic (travel expense reimbursement); stock ownership: Amedica, Axiomed, Benvenue, Expanding Orthopedics, Osprey, Paradigm Spine, Spinal Kinetics, Spineology, Vertiflex. R. A. Lehman: consulting: Medtronic; royalties: Medtronic, Stryker. Z. M. Sardar: consulting: Medtronic; grant/research support from the Department of Defense. J. M. Lombardi: consulting: Medtronic, Stryker. None of the other authors has any conflicts of interest, financial or otherwise, to disclose.

## AUTHOR CONTRIBUTIONS

J.R.M., B.Y., A.M., S.C.K., E.T., O.M., E.S., C.M., K.D.R., N.Y.H., M.S.V., and J.B.C. conceived and designed research; J.R.M., E.F.J., B.Y., J.M.L., Z.M.S., R.A.L., C.M., K.D.R., M.S.V., and J.B.C. performed experiments; J.R.M., E.F.J., J.L.G., and J.B.C. analyzed data; J.R.M., E.F.J., L.M.M., C.M., N.Y.H., M.S.V., and J.B.C. interpreted results of experiments; J.R.M. prepared figures; J.R.M. and J.B.C. drafted manuscript; J.R.M., E.F.J., J.L.G., S.C.K., E.T., O.M., N.Y.H., M.S.V., and J.B.C. edited and revised manuscript; J.R.M., E.F.J., L.M.M., A.M., S.C.K., E.T., O.M., E.S., J.M.L., Z.M.S., R.A.L., C.M., K.D.R., N.Y.H., M.S.V., and J.B.C. approved final version of manuscript.
